# Spontaneous rupture of a renal artery pseudoaneurysm in a previously hypertensive patient

**DOI:** 10.1186/s40885-014-0011-4

**Published:** 2015-01-31

**Authors:** Myung-Sung Kim, Young-Bae Lee, Jae-Hyuk Lee, Chae-Wan Lim, Jun-Hyoung Kim, Hye-Min Choi, Dong-Jin Oh

**Affiliations:** Department of Internal Medicine, Myongji Hospital, 55 Hwasu-ro 14 beon-gil, Deogyang-gu, Goyang 412-826 Korea

**Keywords:** Hypertension, Renal artery, False aneurysm, Spontaneous rupture

## Abstract

Previously, renal artery pseudoaneurysms were thought to be extremely uncommon. However, these lesions are now being detected more frequently as incidental findings on computed tomography, magnetic resonance imaging, and the extensive use of angiography. The incidence of ruptured renal artery pseudoaneurysms is very low. We report a case of a giant renal artery pseudoaneurysm (9.4-cm diameter) with severe left flank pain and a syncopal attack in a young woman who did not control high blood pressure for a couple of years.

## Background

Renal artery pseudoaneurysm is a rare vascular lesion that arises when an arterial injury occurs within the kidney [1]. It is found with increasing frequency as a result of unrelated abdominal imaging or on work-up for hypertension [2]. While rarely symptomatic, it can be a cause of life-threatening hemorrhage and shock [3]. We describe a case of spontaneous rupture, definitely from a pseudoaneurysm of a renal arterial branch, presenting with massive retroperitoneal hemorrhage in a young woman who did not control high blood pressure for a couple of years.

## Case presentation

**Figure 1:**
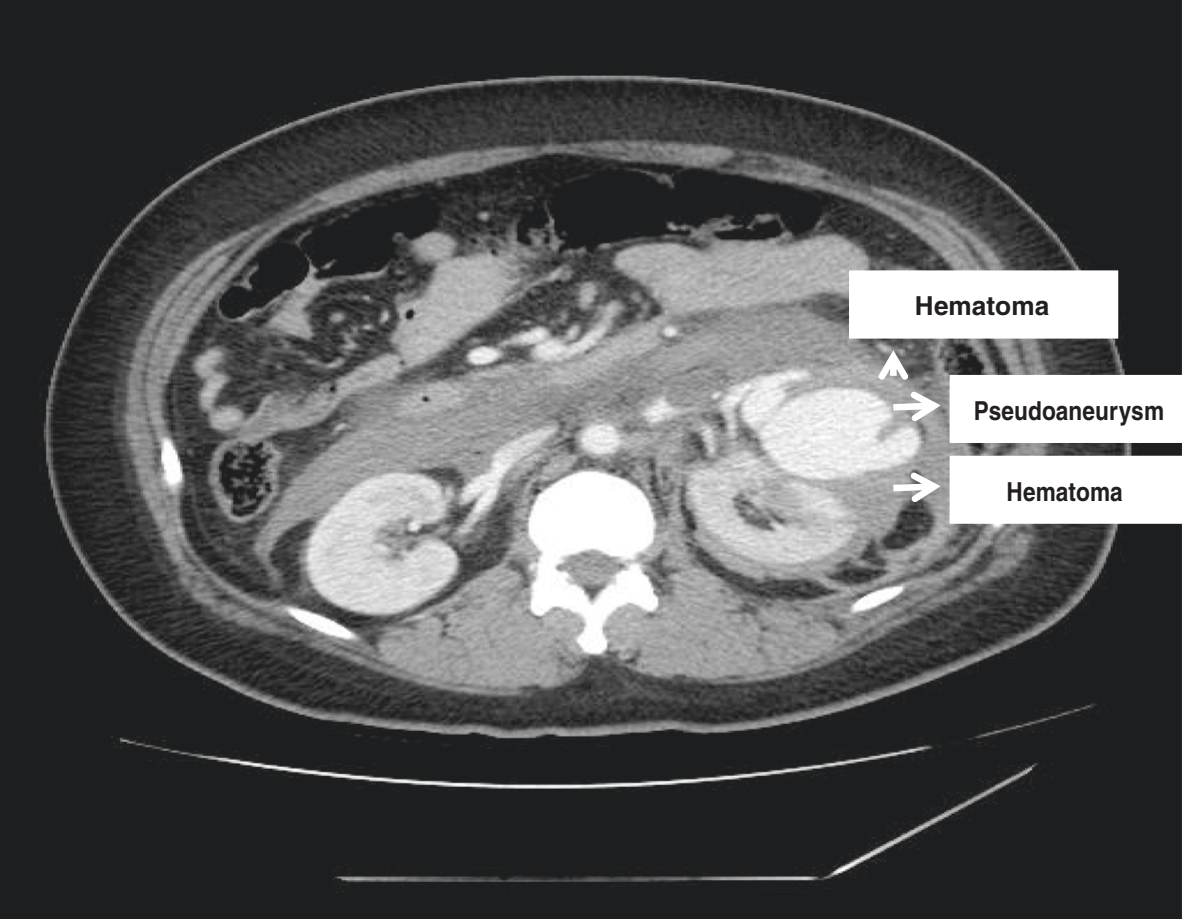
**Computed tomography scan confirmed a large retroperitoneal hematoma from a left renal artery pseudoaneurysm, which extended through a gap in the anterior renal fascia from a left perirenal hematoma.**

**Figure 2:**
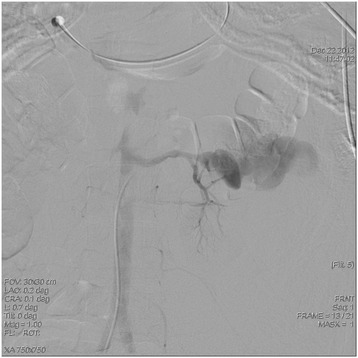
**Left renal angiography showed a pseudoaneurysm on a branch of the left renal artery.** Active bleeding was detected at the time of the angiogram.

**Figure 3:**
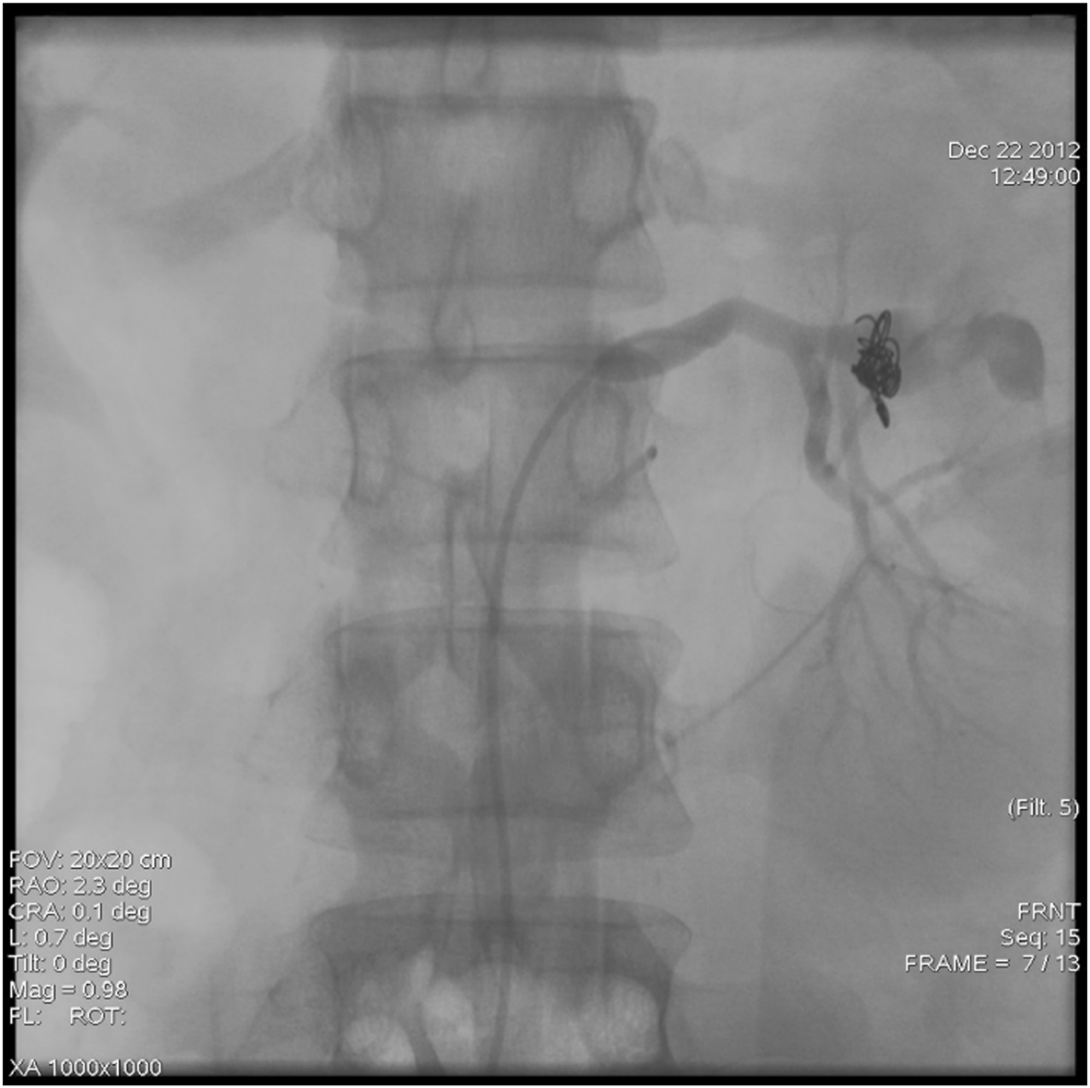
**Embolization was performed using metal coils.**

A 32-year-old woman was admitted with a sudden onset of severe left flank pain. According to history taking, she was hypertensive for a couple of years. However, she did not perform any work-up to elucidate the etiology of high blood pressure. In addition, she did not take any antihypertensive medication despite a local clinic doctor’s recommendation to control high blood pressure. She was not pregnant at admission. There were other specific past medical histories including trauma, renal surgery, percutaneous procedures, as well as inflammatory and neoplastic processes within the kidney. On examination, the patient was in hypovolemic shock (systolic blood pressure, 80 mmHg; hemoglobin, 7.5 g/dL). The abdomen was diffusely tender, guarded, and distended, suggestive of an acute surgical condition. The computed tomography (CT) scan confirmed a large retroperitoneal hematoma from a giant (9.4-cm diameter) left renal artery pseudoaneurysm, which extended through a gap in the anterior renal fascia from a left perirenal hematoma (Figure 1). Active bleeding from the left renal artery pseudoaneurysm was detected at the time of the angiogram (Figure 2). So, we performed embolization using metal coils (Figure 3). The patient had no further episodes of bleeding. After the event, antihypertensive agents using a calcium channel blocker and angiotensin receptor blockade were prescribed to control high blood pressure. Now her blood pressure and renal function are normal.

### Discussion

Renal artery aneurysms including pseudoaneurysms (RAAs) are localized dilations of the renal artery and/or branches. Although rare, there has been a recent increase in the discovery of renal arteriovenous fistulas secondary to trauma, inflammation, renal surgery, and percutaneous needle biopsy [4]. However, more recent literature has demonstrated that the overall incidence ranges between 0.01% and 1% [4,5]. This increases to 2.5% when only patients with hypertension are considered [5] and can be as high as 39% in patients with hypertension unresponsive to medical therapy [6,7]. The mean age at diagnosis is 60 years. RAAs occur more commonly in men and are primarily located on the right [8]. RAAs can be either congenital or acquired. Congenital RAAs have been associated with autosomal dominant polycystic disease, fibromuscular dysplasia, and tuberous sclerosis [9]. Acquired etiologies include long-standing untreated hypertension, atherosclerosis, blunt [4,10] and penetrating trauma, recent surgical manipulation (open, laparoscopic, and/or endovascular) [11], infectious angiomyolipomas (i.e., mycotic) [12], polyarteritis nodosa [8], malignancy, coagulopathy, radiation, and/or cyclophosphamide use [8]. The risk of rupture is thought to vary inversely with size, and most investigators agree that an aneurysm exceeding 2 cm is more likely to undergo rupture. Most RAAs are discovered on a work-up for hypertension (55%) and are more frequently being discovered incidentally during unrelated abdominal imaging (i.e., radiography, color Doppler ultrasound, CT, and magnetic resonance [MR] imaging or angiography) [4]. Although angiography is the gold standard, perhaps the best noninvasive test to evaluate location, size, structure, and relation to nearby organs is CT/MR angiography [8]. When patients present with symptoms, they are usually flank pain and hematuria that can range from mild microscopic hematuria to gross hemorrhage leading to hemodynamic instability [13].

Indications for treatment include hemorrhage, uncontrolled hypertension, pain, progressive enlargement, presence of an arteriovenous fistula, with size >2 to 2.5 cm or size >1 cm in a female of childbearing age [14]. Currently, endovascular surgery is the intervention of choice in elective or emergent circumstances. Accepted endovascular treatments include embolization (i.e., gelfoam, coils, alcohol) or stenting across the aneurysm [15]. There have also been case reports of successful management of RAAs with percutaneous thrombin injection directly into the aneurysm. Factors that may preclude endovascular management are size and multiplicity, although there have been reports of successful endovascular management of large (10 cm) RAAs endovascularly [15], like our case and another case in Korea [16].

## Conclusion

We report a case of a giant renal artery pseudoaneurysm (9.4-cm diameter) with severe left flank pain and a syncopal attack in a young woman who did not control high blood pressure for a couple of years. Therefore, we would like to emphasize the importance of high blood pressure control and the necessity of work-up to elucidate the etiology of high blood pressure especially in patients with newly developed hypertension.

### Consent

Written informed consent was obtained from the patient for publication of this case report and any accompanying images. A copy of the written consent is available for review by the Editor-in-Chief of this journal.

## Abbreviations

CT: Computed tomography
RAAs: Renal artery aneurysms including pseudoaneurysms
MR: Magnetic resonance
