# Erratum To: Spontaneous rupture of a renal artery pseudoaneurysm in a previously hypertensive patient

**DOI:** 10.1186/s40885-015-0024-7

**Published:** 2015-05-29

**Authors:** Myung-Sung Kim, Young-Bae Lee, Jae-Hyuk Lee, Chae-Wan Lim, Jun-Hyoung Kim, Hye-Min Choi, Dong-Jin Oh

**Affiliations:** Department of Internal Medicine, Myoung-Ji Hospital, 55 Hwasu-ro 14beon-gil, Deogyang-gu, Goyang, 412-826 South Korea

After publication of the article [1] it was discovered that a citation error had occurred, resulting in different citation numbers in the PDF and HTML files. The correct citation of 21:4 has now been updated in all versions of the manuscript. We apologise for any inconvenience caused by this error.
